# Differences in Influenza Seasonality by Latitude, Northern India

**DOI:** 10.3201/eid2010.140431

**Published:** 2014-10

**Authors:** Parvaiz A. Koul, Shobha Broor, Siddhartha Saha, John Barnes, Catherine Smith, Michael Shaw, Mandeep Chadha, Renu B. Lal

**Affiliations:** Sheri-Kashmir Institute of Medical Sciences, Srinagar, India (P.A. Koul);; All India Institute of Medical Sciences, New Delhi, India (S. Broor);; Centers for Disease Control and Prevention, Atlanta, Georgia, USA (S. Saha, J. Barnes, C. Smith, M. Shaw, R.B. Lal);; National Institute of Virology, Pune, India (M. Chadha)

**Keywords:** seasonal influenza, India, pandemic influenza, influenza, viruses, latitude, geography, seasonality, New Delhi, Srinagar

## Abstract

The seasonality of influenza in the tropics complicates vaccination timing. We investigated influenza seasonality in northern India and found influenza positivity peaked in Srinagar (34.09°N) in January–March but peaked in New Delhi (28.66°N) in July–September. Srinagar should consider influenza vaccination in October–November, but New Delhi should vaccinate in May–June.

Annual and pandemic influenza are sources of considerable illness and death worldwide (*1*). Human influenza infection rates peak during the winter months in temperate regions; however, the pattern of influenza is different in tropical and subtropical areas, with year-round circulation in some areas and biannual peaks in others (*2*–*5*). The complex seasonality of influenza in the tropics complicates appropriate vaccination recommendations, particularly the timing of vaccination campaigns in tropical regions (*3*,*4*).

India has discrete seasons that vary greatly from north to south. Jammu and Kashmir, the northernmost state of India, has severe winters from December to March, whereas Delhi, the capital region, has milder winters. Sentinel surveillance for influenza in India has shown distinct influenza peaks in India (*6*–*8*). We undertook influenza surveillance during 2011–2012 in 2 cities in northern India, Srinagar and New Delhi, which are ≈500 km apart, and found evidence for discrete seasonality related to the latitudes of these cities, a finding that has implications for influenza vaccination policy and timing.

## The Study

For the study, we enrolled patients who attended the outpatient clinics at the All India Institute of Medical Sciences, New Delhi, and Sheri-Kashmir Institute of Medical Sciences, Srinagar, during 2011–2012 who had influenza-like illness (ILI; defined as sudden onset or history of fever >38°C, cough or sore throat and/or rhinorrhea) (*6*). We collected 5–10 nasopharyngeal samples from these patients each week and tested them for influenza viruses by type and subtype (*9*,*10*). A confirmed case-patient was defined as a patient meeting the ILI case definition who had positive results for influenza by reverse transcription PCR. Sanger sequencing of the hemagglutinin and neuraminidase genes was completed, and consensus was used to construct a Kimura 2-parameter neighbor-joining tree (Technical Appendix Figure 1). Sequences from New Delhi and Srinagar were compared with those of vaccine strains and with published cognate sequences of corresponding genes (Technical Appendix Table 1), including those from India (*10*).

**Figure 1:**
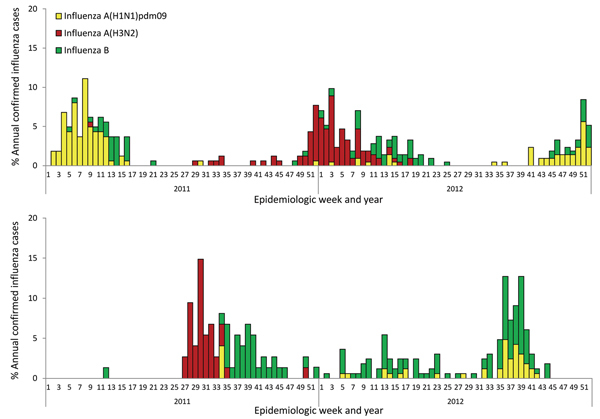
Weekly trends and proportion of annual numbers of positive influenza cases, by epidemiologic week and influenza type, Srinagar (A) and New Delhi (B), India, 2011–2012. Clear seasonal peaks are seen in January–March (weeks 1–16) for Srinagar and in July and September (weeks 28–40) for New Delhi.

Surveillance for influenza viruses revealed that overall influenza positivity was 17.6% (375 confirmed cases from 2,126 ILI patients) in Srinagar and 9.46% (239/2,526) in New Delhi (Table). Discrete winter time peaks were observed during January–March (epidemiologic weeks [EW] 1–12) in Srinagar, whereas New Delhi had peaks of influenza circulation during July 2011 and September 2012 (EW 26–36) (Figure 1). Influenza A was the predominant type in Srinagar (275/375; 72.9%), whereas influenza B dominated in New Delhi (154/239; 64.4%). Circulation of influenza A(H3N2) during the monsoon season of 2011 in New Delhi was followed by predominance of H3N2 during winter 2012 in Srinagar (Figure 1).

**Table:** Influenza surveillance, Srinagar and Delhi, India, 2011–2012

City	No. (%) persons		
	2011	2012	Total
Srinagar (34.09°N)			
Tested	768	1,371	2,139
Influenza positive	162 (21)	215 (15.7)	377 (17.6)
Influenza type*			
A(H1N1)pdm09	95 (58.6)	57 (26.5)	152 (40.3)
A(H3N2)	38 (23.5)	85 (39.5)	123 (32.6)
B	29 (17.9)	73 (34.0)	102 (27.1)
New Delhi (28.66°N)			
Tested	1,007	1,519	2,526
Influenza positive	74 (7.3)	165 (10.8)	239 (9.46)
Influenza type*			
A(H1N1)pdm09	3 (4.1)	44 (26.7)	47 (19.7)
A(H3N2)	38 (51.4)	0	38 (15.9)
B	33 (44.6)	121 (73.3)	154 (64.4)

*Percentages are of all influenza-positive test results in category.

Phylogenetic analysis of the hemagglutinin and neuraminidase genes from selected samples from New Delhi and Srinagar revealed no notable differences between circulating viruses (Technical Appendix Figure 1). Furthermore, circulating influenza strains were closely related to the selected influenza vaccine strains, A/California/7/2009 (H1N1), A/Perth/16/2009 (H3N2), and B/Brisbane/60/2008, which remained unchanged for 2011–2012 for the Northern and Southern Hemisphere formulations (Technical Appendix Figure 2). The 2012–2013 Northern Hemisphere formulation changed the H3N2 strain to A/Victoria/361/2011 and the influenza B strain to B/Wisconsin/1/2010, but sequence information from 2013 circulating viruses from Srinagar was not available to assess vaccine similarity.

**Figure 2:**
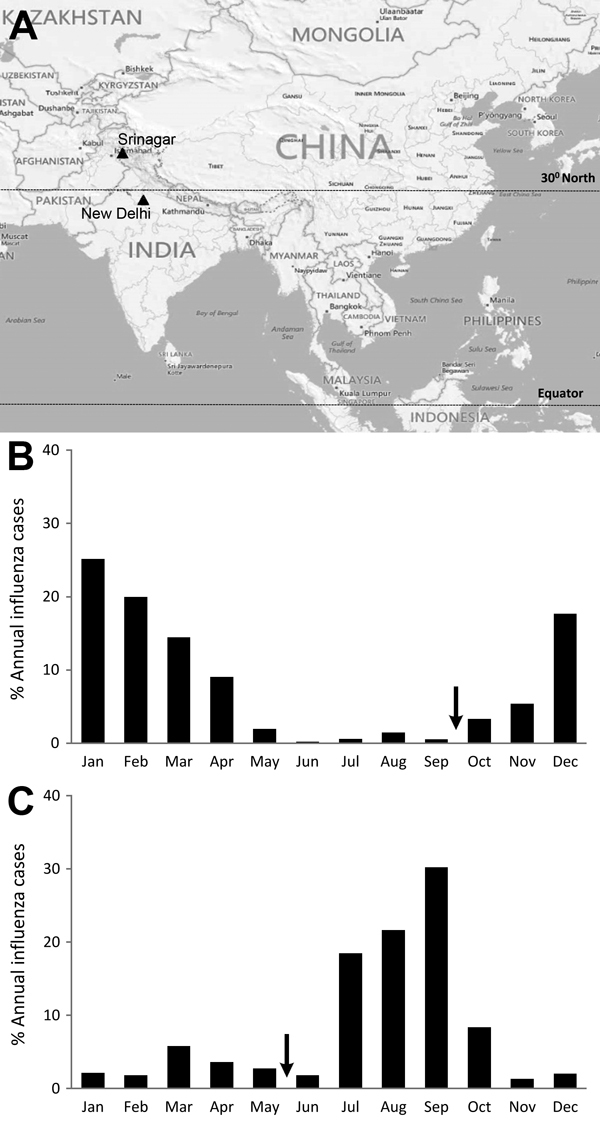
Comparison of latitudes of Srinagar and New Delhi, India, and distribution of influenza virus infections, 2011–2012. A) Locations of Srinagar and New Delhi (black triangles), with vertical lines indicating 30°N latitude and equator. B) Monthly distribution of cases of influenza virus infection in Srinagar (34.0°N latitude). C) Monthly distribution of cases of influenza virus infection in New Delhi (28.7°N latitude). Arrows indicate proposed vaccination timing; latitude of each city is shown. Map created in Epi-Map in Epi Info 7 (Centers for Disease Control and Prevention, Atlanta, GA, USA).

Analysis of meteorologic factors (i.e., rainfall, temperature, relative humidity, vapor pressure, and dew point) showed that the monthly proportion of influenza positivity correlated with decreased temperature and dew point in Srinagar and with rainfall amount in New Delhi (data not shown). Cumulative data over the 2-year surveillance period revealed differences in seasonality by latitude; influenza positivity peaked during December–February in Srinagar (34.09°N) but in July–September in New Delhi (28.66°N) (Figure 2). Influenza seasonality indicates that New Delhi would likely benefit from springtime vaccination (May–June), whereas vaccination in the fall (October–November) would be better for Srinagar (Figure 2). We recently illustrated that India and most other tropical countries in Asia exhibit influenza seasonality that coincides with the monsoon season, June–October (*11*).

## Conclusions

We identified discrete patterns of influenza circulation in India. In Srinagar, a city in the northernmost region of India, influenza positivity rates peaked in winter (December–March), whereas in New Delhi, a city just ≈500 km south of Srinagar, influenza peaked during the monsoon season (July–September). The winter peak in Srinagar is similar to the timing of influenza circulation observed for most countries in Europe and United States in the Northern Hemisphere (*4*,*5*). By contrast, the data on influenza seasonality in New Delhi corroborate findings which showed that many countries in tropical regions (e.g., Brazil, India, Vietnam) experience high influenza transmission rates during the rainy season (*6*,*11*–*13*). Overall, influenza A and B viruses co-circulated throughout the surveillance period in Srinagar and New Delhi; however, the types and subtypes varied.

Peaks of influenza circulation in Srinagar and New Delhi show seasonal patterns that depend on factors such as temperature, rainfall, humidity, and latitude (*2*,*3*). Srinagar, at >30°N latitude, has influenza seasonality that coincides with lower temperature and low dew points during winter, whereas New Delhi, at <30°N latitude, has a peak of influenza circulation that coincides with the rainy season. Seasonal influenza activity coinciding with the humid, rainy season at lower latitudes has also been observed in large areas of Central and South America and southern Asia (*11*,*13*). In contrast, cold, dry weather was predictive of influenza peaks at higher latitudes, as observed for Srinagar. The latitude dependence of influenza circulation observed in this study is similar to such dependence observed in Brazil and China (*13*,*14*) and collectively suggests that decisions on the timing of influenza vaccination should not be based only on the hemisphere a country is in but also on the types of seasonal patterns that exist within a country (*15*). These latitudinal differences in influenza seasonality in India have implications for influenza vaccine timing and vaccine formulation.

Influenza vaccine induces a neutralizing antibody response that wanes over time. Thus, the timing of vaccination has a direct effect on vaccine effectiveness. In the northernmost part of India, peak influenza circulation occurs during the winter months; therefore, vaccination during October–November using the Northern Hemisphere vaccine formulation may be appropriate. However, this practice would not be appropriate in the Delhi metropolitan region, where influenza peaks in July–September. In addition, whereas our data points to an approximate latitude where temperate and tropical patterns for influenza peaks diverge, more robust data with multiple surveillance sites in tropical, subtropical, and temperate regions in India and China are needed to define the exact latitude points for influenza circulation patterns.

Our study has limitations. First, comparative data were available only for 2 years. Additional surveillance data for multiple years and many cities around the latitude gradient are required to further corroborate these observations. Furthermore, validation of vaccine formulation will require tracking of additional circulating influenza strains over several epidemic periods.

In summary, we identified 2 distinct seasons for influenza circulation in 2 cities in India. We recommend that policy makers in India review circulation patterns closely before implementing influenza intervention plans. Our data suggest that cities in India located north of 30°N latitude can continue vaccination in the winter, but those south of 30°N, including New Delhi, should consider vaccination in May–June (*15*). Collectively, these data should help decision makers, especially regulatory authorities, choose vaccines and vaccination schedules best suited for each region.

Technical Appendix Accession numbers of isolates used for phylogenetic analysis, phylogenetic analysis of hemagglutinin and neuraminidase sequences of influenza virus strains from Srinagar and New Delhi, India, compared with published sequences and corresponding vaccine strains, and World Health Organization recommended vaccines by season and hemisphere, 2006–2014.

## References

[1] Simonsen L. The global impact of influenza on morbidity and mortality. Vaccine. 1999;17(Suppl 1):S3–10 . 10.1016/S0264-410X(99)00099-7 10471173

[2] Tamerius JD, Shaman J, Alonso WJ, Bloom-Feshbach K, Uejio CK, Comrie A, Environmental predictors of seasonal influenza epidemics across temperate and tropical climates. PLoS Pathog. 2013;9:e1003194. 10.1371/journal.ppat.1003194 23505366 PMC3591336

[3] Azziz Baumgartner E, Dao CN, Nasreen S, Bhuiyan MU, Mah EMS, Al Mamun A, Seasonality, timing, and climate drivers of influenza activity worldwide. J Infect Dis. 2012;206:838–46. 10.1093/infdis/jis467 22829641

[4] Viboud C, Alonso WJ, Simonsen L. Influenza in tropical regions. PLoS Med. 2006;3:e89 . 10.1371/journal.pmed.0030089 16509764 PMC1391975

[5] Park AW, Glass K. Dynamic patterns of avian and human influenza in east and southeast Asia. Lancet Infect Dis. 2007;7:543–8. 10.1016/S1473-3099(07)70186-X 17646027

[6] Chadha MS, Broor S, Gunasekaran P, Potdar VA, Krishnan A, Chawla-Sarkar M, Multisite virological influenza surveillance in India: 2004–2008. Influenza Other Respir Viruses. 2012;6:196–203. 10.1111/j.1750-2659.2011.00293.x PMC5657135 21955356

[7] Broor S, Krishnan A, Roy DS, Dhakad S, Kaushik S, Mir MA, Dynamic patterns of circulating seasonal and pandemic A(H1N1)pdm09 influenza viruses from 2007–2010 in and around Delhi, India. PLoS ONE. 2012;7:e29129. 10.1371/journal.pone.0029129 22235265 PMC3250412

[8] Koul PA, Mir MA, Bali NK, Chawla-Sarkar M, Sarkar M, Kaushik S, Pandemic and seasonal influenza viruses among patients with acute respiratory illness in Kashmir (India). Influenza Other Respir Viruses. 2011;5:e521. 10.1111/j.1750-2659.2011.00261.x PMC5780669 21668665

[9] Centers for Disease Control and Prevention. CDC protocol of realtime RTPCR for swine influenza A(H1N1). 2009 Apr 28 [cited 2013 Jun 25]. http://www.who.int/csr/resources/publications/swineflu/CDCrealtimeRTPCRprotocol_20090428.pdf

[10] Potdar VA, Chadha MS, Jadhav SM, Mullick J, Cherian SS, Mishra AC. Genetic characterization of the influenza A pandemic (H1N1) 2009 virus isolates from India. PLoS ONE. 2010;5:e9693. 10.1371/journal.pone.0009693 20300625 PMC2837743

[11] Saha S, Chadha M, Mamun AA, Rahman M, Sturm-Ramirez K, Chittaganpitch M, Influenza seasonality and vaccination timing in tropics and subtropics of south and south-east Asia. Bull World Health Organ. 2014;92:318–30. 10.2471/BLT.13.124412 24839321 PMC4007122

[12] Western Pacific Region Global Influenza Surveillance and Response System. Epidemiological and virological characteristics of influenza in the Western Pacific Region of the World Health Organization, 2006–2010. PLoS ONE. 2012;7:e37568. 10.1371/journal.pone.0037568 22675427 PMC3366627

[13] Moura FE, Perdigao AC, Siqueira MM. Seasonality of influenza in the tropics: a distinct pattern in northeastern Brazil. Am J Trop Med Hyg. 2009;81:180–3. 19556586

[14] Yu H, Alonso WJ, Feng L, Tan Y, Shu Y, Yang W, Characterization of regional influenza seasonality patterns in china and implications for vaccination strategies: spatio-temporal modeling of surveillance data. PLoS Med. 2013;10:e1001552. 10.1371/journal.pmed.1001552 24348203 PMC3864611

[15] Cox N. Influenza seasonality: timing and formulation of vaccines. Bull World Health Organ. 2014;92:311. 10.2471/BLT.14.139428 24839317 PMC4007136

