# Supplementary material for: Differences in Influenza Seasonality by Latitude, Northern India
Source: Emerg Infect Dis. 2014 Oct;20(10):1723–6. doi: 10.3201/eid2010.140431 (PMC4193176; doi:10.3201/eid2010.140431)
Supplement: Technical Appendix — Accession numbers of isolates used for phylogenetic analysis, phylogenetic analysis of hemagglutinin and neuraminidase sequences of influenza virus strains from Srinagar and New Delhi, India, compared with published sequences and corresponding vaccine strains, and World Health Organization recommended vaccines by season and hemisphere, 2006–2014. [file 14-0431-Techapp-s1.pdf]

Article DOI: http://dx.doi.org/10.3201/eid2010.140431

Differences in Influenza Seasonality by Latitude, Northern India

Technical Appendix

Technical Appendix Table. Influenza strains and gene segments from GISAID EpiFlu database used in this study*

EPI-Segment-ID	Segment	Length	Strain
EPI332607	HA	1701	A/Alaska/14/2011
EPI215877	NA	1410	A/Alaska/14/2011
EPI325551	HA	1701	A/American Samoa/4520/2011
EPI325575	NA	1410	A/American Samoa/4520/2011
EPI404814	HA	1701	A/Argentina/1033/2012
EPI331203	NA	1410	A/Argentina/1033/2012
EPI267876	HA	1701	A/Argentina/19527/2009
EPI331206	NA	1410	A/Argentina/19527/2009
EPI331198	HA	1701	A/Argentina/63/2011
EPI273913	NA	1410	A/Argentina/63/2011
EPI335786	HA	1701	A/Argentina/8836/2011
EPI319996	NA	1410	A/Argentina/8836/2011
EPI330987	HA	1701	A/Arkansas/03/2011
EPI215722	NA	1410	A/Arkansas/03/2011
EPI331201	HA	1701	A/Bangladesh/8324/2011
EPI273910	NA	1410	A/Bangladesh/8324/2011
EPI215724	NA	1410	A/Barbados/104/2011
EPI194113	HA	1701	A/Bolivia/1263/2009
EPI319991	NA	1410	A/Bolivia/1263/2009
EPI341964	HA	1701	A/Bolivia/193/2011
EPI320120	NA	1410	A/Bolivia/193/2011
EPI404834	HA	1701	A/Bolivia/3040/2012
EPI325559	NA	1410	A/Bolivia/3040/2012
EPI280335	HA	1701	A/Brisbane/10/2010
EPI273896	NA	1410	A/Brisbane/10/2010
EPI273609	HA	1701	A/California/07/2009
EPI325587	NA	1410	A/California/07/2009
EPI316335	HA	1701	A/California/17/2011
EPI331040	HA	1701	A/Cape Town/60/2011
EPI325572	NA	1410	A/Cape Town/60/2011
EPI273914	HA	1701	A/Castro/12274/2010
EPI331209	NA	1410	A/Castro/12274/2010
EPI186253	HA	1701	A/Chile/1174/2009
EPI325566	NA	1410	A/Chile/1174/2009
EPI325567	HA	1701	A/Chile/293/2011
EPI331041	NA	1410	A/Chile/293/2011
EPI280341	HA	1701	A/Christchurch/16/2010
EPI325578	NA	1410	A/Christchurch/16/2010
EPI335801	HA	1701	A/Concepcion/16695/2011
EPI331215	NA	1410	A/Concepcion/16695/2011
EPI397078	NA	1410	A/Connecticut/08/2011
EPI537424	NA	1410	A/Delhi/1717/2012
EPI537425	NA	1410	A/Delhi/1938/2012
EPI537426	NA	1410	A/Delhi/1939/2012
EPI537428	HA	1701	A/Delhi/1966/2012
EPI537427	NA	1410	A/Delhi/1966/2012
EPI320011	HA	1701	A/Dominican Republic/5059/2011
EPI370247	NA	1410	A/Dominican Republic/5059/2011
EPI271947	NA	1410	A/Dominican Republic/5145/2011
EPI325588	HA	1701	A/Federated States Of Micronesia/6119/2011
EPI335800	NA	1410	A/Federated States Of Micronesia/6119/2011
EPI370248	HA	1701	A/Fiji/2/2012
EPI320010	NA	1410	A/Fiji/2/2012
EPI215725	HA	1701	A/Fiji/2048/2009

EPI-Segment-ID	Segment	Length	Strain
EPI331218	NA	1410	A/Fiji/2048/2009
EPI342420	HA	1701	A/Florida/16/2010
EPI341963	NA	1410	A/Florida/16/2010
EPI331204	HA	1701	A/Florida/19/2011
EPI186252	NA	1410	A/Florida/19/2011
EPI379530	HA	1701	A/Goroka/16/2011
EPI320013	NA	1410	A/Goroka/16/2011
EPI325576	HA	1701	A/Heilongjiang-Xiangfang/191/2011
EPI426452	NA	1410	A/Heilongjiang-Xiangfang/191/2011
EPI294253	NA	1410	A/Hong Kong/3962/2011
EPI331207	HA	1701	A/Hong Kong/3973/2011
EPI404813	NA	1410	A/Hong Kong/3973/2011
EPI194114	NA	1410	A/Hunan-Jishou/1116/2011
EPI278875	HA	1701	A/India/007/2010
EPI254552	NA	1410	A/India/007/2010
EPI227676	HA	1701	A/India/2993/2009
EPI280334	NA	1410	A/India/2993/2009
EPI295462	HA	1701	A/India/3508/2010
EPI353908	NA	1410	A/India/3508/2010
EPI273897	HA	1701	A/India/3725/2010
EPI227675	NA	1410	A/India/3725/2010
EPI254551	HA	1701	A/India/4725/2009
EPI342419	NA	1410	A/India/4725/2009
EPI325579	HA	1701	A/India/4947/2011
EPI267878	NA	1410	A/India/4947/2011
EPI295459	HA	1701	A/India/5103/2010
EPI278870	NA	1410	A/India/5103/2010
EPI278871	HA	1701	A/India/5107/2010
EPI232917	NA	1410	A/India/5107/2010
EPI353396	HA	1701	A/India/5756/2011
EPI272731	NA	1410	A/India/5756/2011
EPI232918	HA	1701	A/India/8489/2009
EPI280340	NA	1410	A/India/8489/2009
EPI278873	HA	1701	A/India/8910/2010
EPI295458	NA	1410	A/India/8910/2010
EPI280325	NA	1410	A/India/8942/2012
EPI397079	HA	1701	A/Iquique/50771/2012
EPI325584	NA	1410	A/Iquique/50771/2012
EPI286990	HA	1701	A/Johannesburg/115/2010
EPI332584	NA	1410	A/Johannesburg/115/2010
EPI342434	HA	1701	A/Kentucky/09/2010
EPI409911	NA	1410	A/Kentucky/09/2010
EPI215723	HA	1701	A/Kiribati/2003/2009
EPI386048	NA	1410	A/Kiribati/2003/2009
EPI390481	HA	1701	A/Madagascar/00695/2012
EPI332597	NA	1410	A/Madagascar/00695/2012
EPI243975	NA	1410	A/Madagascar/10201/2009
EPI309970	HA	1701	A/Maryland/04/2011
EPI278872	NA	1410	A/Maryland/04/2011
EPI331210	HA	1701	A/Mexico/2208/2011
EPI387971	NA	1410	A/Mexico/2208/2011
EPI310015	HA	1701	A/Minnesota/03/2011
EPI278874	NA	1410	A/Minnesota/03/2011
EPI273850	NA	1410	A/Minnesota/11/2011
EPI215717	HA	1701	A/Nauru/2008/2009
EPI273608	NA	1410	A/Nauru/2008/2009
EPI349252	HA	1701	A/New Caledonia/11/2011
EPI309969	NA	1410	A/New Caledonia/11/2011
EPI320095	HA	1701	A/New Mexico/07/2011
EPI194243	NA	1410	A/New Mexico/08/2011
EPI331213	HA	1701	A/New York/09/2011
EPI349351	NA	1410	A/New York/09/2011
EPI332598	HA	1701	A/Nigeria/4280/2011
EPI404833	NA	1410	A/Nigeria/4280/2011
EPI332585	HA	1701	A/Nonthaburi/78/2011
EPI335785	NA	1410	A/Nonthaburi/78/2011
EPI325560	HA	1701	A/Ontario/RV1255/2011
EPI286989	NA	1410	A/Ontario/RV1255/2011
EPI215653	HA	1701	A/Palau/1/2009

EPI-Segment-ID	Segment	Length	Strain
EPI310014	NA	1410	A/Palau/1/2009
EPI387972	HA	1701	A/Paraguay/114/2012
EPI316434	NA	1410	A/Paraguay/114/2012
EPI349352	HA	1701	A/Paraguay/191/2011
EPI316438	NA	1410	A/Paraguay/191/2011
EPI301698	HA	1701	A/Paraguay/813/2010
EPI316467	NA	1410	A/Paraguay/813/2010
EPI320121	HA	1701	A/Pennsylvania/02/2011
EPI272299	NA	1410	A/Pennsylvania/02/2011
EPI409912	HA	1701	A/Peru/0429/2012
EPI320094	NA	1410	A/Peru/0429/2012
EPI243881	HA	1701	A/Peru/8109/2009
EPI331212	NA	1410	A/Peru/8109/2009
EPI273911	HA	1701	A/Puerto Montt/11868/2010
EPI316334	NA	1410	A/Puerto Montt/11868/2010
EPI233072	HA	1701	A/Samoa/48/2009
EPI331197	NA	1410	A/Samoa/48/2009
EPI335825	HA	1701	A/Santiago/14453/2011
EPI320084	NA	1410	A/Santiago/14453/2011
EPI325573	HA	1701	A/Santiago/7774/2011
EPI390482	NA	1410	A/Santiago/7774/2011
EPI194242	HA	1701	A/Seychelles/106/2009
EPI320097	NA	1410	A/Seychelles/106/2009
EPI271948	HA	1701	A/Simbu/1/2010
EPI319998	NA	1410	A/Simbu/1/2010
EPI210545	NA	1410	A/South Africa/2/2009
EPI272732	HA	1701	A/South Carolina/02/2010
EPI342433	NA	1410	A/South Carolina/02/2010
EPI537430	HA	1701	A/Srinigar/04/2011
EPI537429	NA	1410	A/Srinigar/04/2011
EPI537444	HA	1701	A/Srinigar/1118/2011
EPI537443	NA	1410	A/Srinigar/1118/2011
EPI537446	HA	1701	A/Srinigar/1186/2011
EPI537445	NA	1410	A/Srinigar/1186/2011
EPI537434	HA	1701	A/Srinigar/126/2011
EPI537433	NA	1410	A/Srinigar/126/2011
EPI537432	HA	1701	A/Srinigar/201/2011
EPI537431	NA	1410	A/Srinigar/201/2011
EPI537435	NA	1410	A/Srinigar/226/2011
EPI537437	HA	1701	A/Srinigar/234/2011
EPI537436	NA	1410	A/Srinigar/234/2011
EPI537439	HA	1701	A/Srinigar/385/2011
EPI537438	NA	1410	A/Srinigar/385/2011
EPI537440	NA	1410	A/Srinigar/410/2011
EPI537442	HA	1701	A/Srinigar/827/2011
EPI537441	NA	1410	A/Srinigar/827/2011
EPI316435	HA	1701	A/St. Petersburg/100/2011
EPI233071	NA	1410	A/St. Petersburg/100/2011
EPI215652	NA	1410	A/St. Petersburg/25/2011
EPI440929	HA	1701	A/Tanzania/2131/2012
EPI320021	NA	1410	A/Tanzania/2131/2012
EPI273851	HA	1701	A/Tanzania/233/2010
EPI332609	NA	1410	A/Tanzania/233/2010
EPI331216	HA	1701	A/Texas/10/2011
EPI301697	NA	1410	A/Texas/10/2011
EPI331219	HA	1701	A/Uganda/2707/2011
EPI243880	NA	1410	A/Uganda/2707/2011
EPI294254	HA	1701	A/Vanimo/7/2010
EPI342433	NA	1410	A/Vanimo/7/2010
EPI316468	HA	1701	A/Voronezh/1/2011
EPI215716	NA	1410	A/Voronezh/1/2011
EPI280326	HA	1701	A/Wisconsin/08/2010
EPI295461	NA	1410	A/Wisconsin/08/2010
EPI215878	HA	1701	A/Yap/2020/2009
EPI325544	NA	1410	A/Yap/2020/2009
EPI272184	HA	1701	A/Zambia/CZC1/2009
EPI331200	NA	1410	A/Zambia/CZC1/2009

*http://www.gisaid.org.

Technical Appendix Figure 1 (following pages). Phylogenetic analysis of hemagglutinin (HA) and neuraminidase (NA) gene sequences of influenza virus strains from Srinagar and New Delhi, India, compared with published sequences and corresponding vaccine strains. Strains from Srinagar are shown in blue, from Delhi in red, and from influenza vaccine in purple; other strains are from published sequences, including those from India. A) Influenza A(H1N1)pdm09 HA gene; B) influenza A(H1N1)pdm09 NA gene; C) influenza A(H3N2) HA gene; D) influenza A(H3N2) NA gene; E) influenza B Victoria lineage HA gene; F) influenza B Victoria lineage NA gene; G) influenza B Yamagata lineage HA gene; and H) influenza B Yamagata lineage NA gene. HA-1 and NA genes were amplified and PCR products were sequenced using the dideoxynucleotides chain termination method using Big-Dye Terminator Chemistry (Life Technologies, Grand Island, NY, USA). A neighbor-joining tree was generated by using pairwise gap deletion and maximum composite likelihood using the Tamura-Nei nucleotide model in MEGA4 (http://www.megasoftware.net).

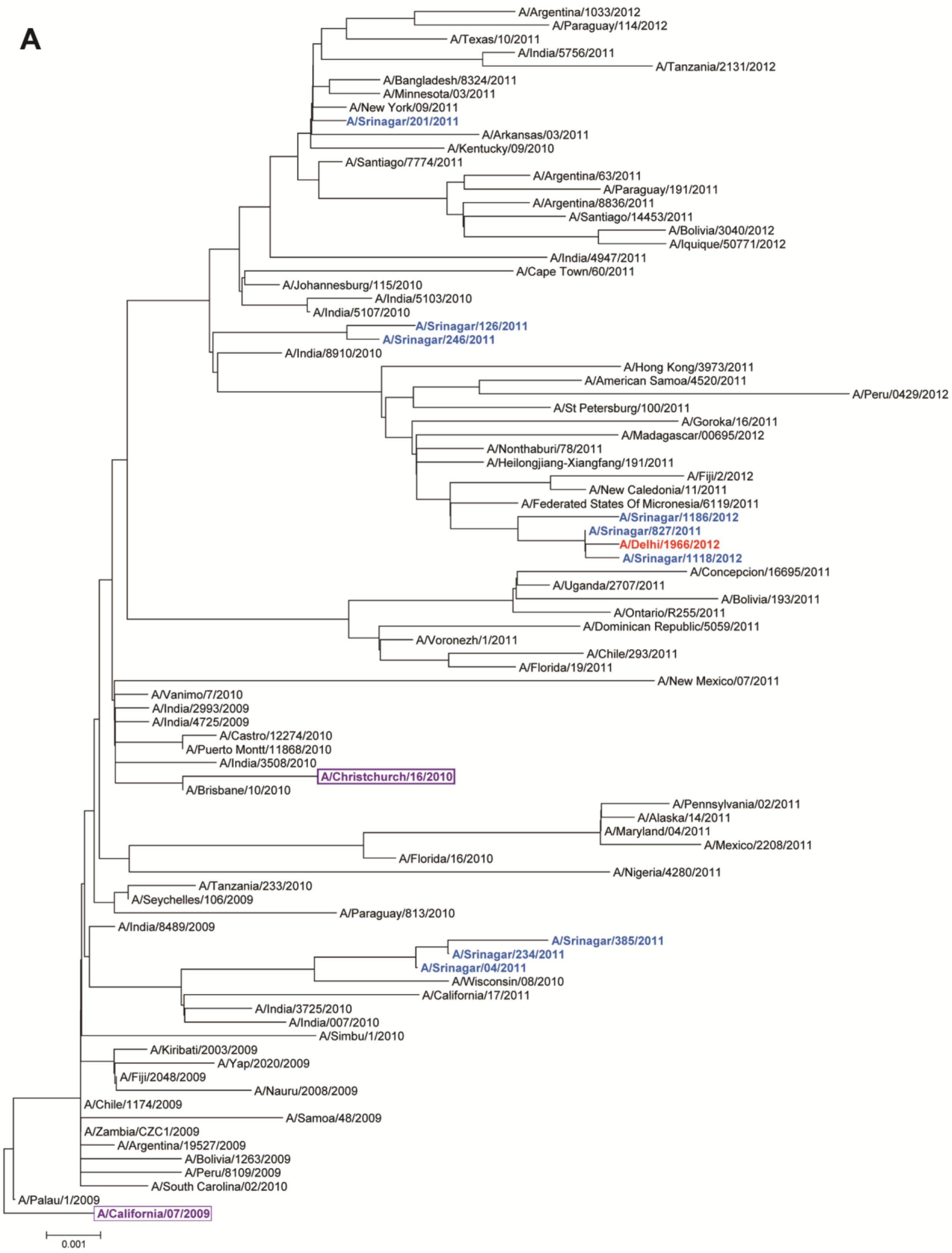
A
A/Argentina/1033/2012
A/Paraguay/114/2012
A/Texas/10/2011
A/India/5756/2011
A/Tanzania/2131/2012
A/Bangladesh/8324/2011
A/Minnesota/03/2011
A/New York/09/2011
A/Srinagar/201/2011
A/Arkansas/03/2011
A/Kentucky/09/2010
A/Santiago/7774/2011
A/Argentina/63/2011
A/Paraguay/191/2011
A/Argentina/8836/2011
A/Santiago/14453/2011
A/Bolivia/3040/2012
A/Iquique/50771/2012
A/India/4947/2011
A/Cape Town/60/2011
A/Johannesburg/115/2010
A/India/5103/2010
A/India/5107/2010
A/Srinagar/126/2011
A/Srinagar/246/2011
A/India/8910/2010
A/Hong Kong/3973/2011
A/American Samoa/4520/2011
A/Peru/0429/2012
A/St Petersburg/100/2011
A/Goroka/16/2011
A/Madagascar/00695/2012
A/Nonthaburi/78/2011
A/Heilongjiang-Xiangfang/191/2011
A/Fiji/2/2012
A/New Caledonia/11/2011
A/Federated States Of Micronesia/6119/2011
A/Srinagar/1186/2012
A/Srinagar/827/2011
A/Delhi/1966/2012
A/Srinagar/1118/2012
A/Concepcion/16695/2011
A/Uganda/2707/2011
A/Bolivia/193/2011
A/Ontario/R255/2011
A/Dominican Republic/5059/2011
A/Voronezh/1/2011
A/Chile/293/2011
A/Florida/19/2011
A/New Mexico/07/2011
A/Vanimo/7/2010
A/India/2993/2009
A/India/4725/2009
A/Castro/12274/2010
A/Puerto Montt/11868/2010
A/India/3508/2010
A/Christchurch/16/2010
A/Brisbane/10/2010
A/Pennsylvania/02/2011
A/Alaska/14/2011
A/Maryland/04/2011
A/Mexico/2208/2011
A/Florida/16/2010
A/Nigeria/4280/2011
A/Tanzania/233/2010
A/Seychelles/106/2009
A/Paraguay/813/2010
A/India/8489/2009
A/Srinagar/385/2011
A/Srinagar/234/2011
A/Srinagar/04/2011
A/Wisconsin/08/2010
A/California/17/2011
A/India/3725/2010
A/India/007/2010
A/Simbu/1/2010
A/Kiribati/2003/2009
A/Yap/2020/2009
A/Fiji/2048/2009
A/Nauru/2008/2009
A/Chile/1174/2009
A/Samoa/48/2009
A/Zambia/CZC1/2009
A/Argentina/19527/2009
A/Bolivia/1263/2009
A/Peru/8109/2009
A/South Carolina/02/2010
A/Palau/1/2009
A/California/07/2009
0.001

B

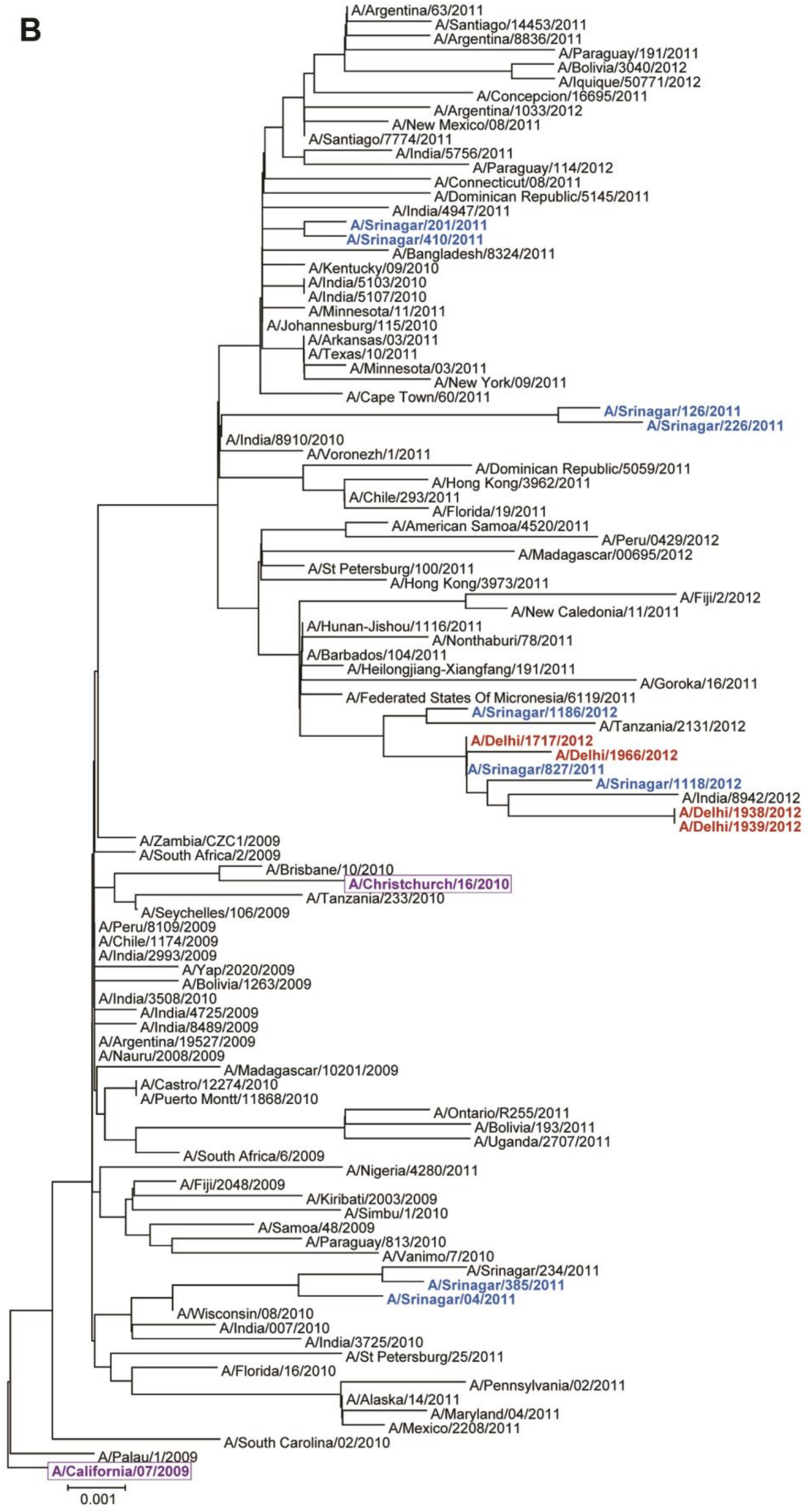
A/Argentina/63/2011
A/Santiago/14453/2011
A/Argentina/8836/2011
A/Paraguay/191/2011
A/Bolivia/3040/2012
A/Iquique/50771/2012
A/Concepcion/16695/2011
A/Argentina/1033/2012
A/New Mexico/08/2011
A/Santiago/7774/2011
A/India/5756/2011
A/Paraguay/114/2012
A/Connecticut/08/2011
A/Dominican Republic/5145/2011
A/India/4947/2011
A/Srinagar/201/2011
A/Srinagar/410/2011
A/Bangladesh/8324/2011
A/Kentucky/09/2010
A/India/5103/2010
A/India/5107/2010
A/Minnesota/11/2011
A/Johannesburg/115/2010
A/Arkansas/03/2011
A/Texas/10/2011
A/Minnesota/03/2011
A/New York/09/2011
A/Cape Town/60/2011
A/Srinagar/126/2011
A/Srinagar/226/2011
A/India/8910/2010
A/Voronezh/1/2011
A/Dominican Republic/5059/2011
A/Hong Kong/3962/2011
A/Chile/293/2011
A/Florida/19/2011
A/American Samoa/4520/2011
A/Peru/0429/2012
A/Madagascar/00695/2012
A/St Petersburg/100/2011
A/Hong Kong/3973/2011
A/Fiji/2/2012
A/New Caledonia/11/2011
A/Hunan-Jishou/1116/2011
A/Nonthaburi/78/2011
A/Barbados/104/2011
A/Heilongjiang-Xiangfang/191/2011
A/Goroka/16/2011
A/Federated States Of Micronesia/6119/2011
A/Srinagar/1186/2012
A/Tanzania/2131/2012
A/Delhi/1717/2012
A/Delhi/1966/2012
A/Srinagar/827/2011
A/Srinagar/1118/2012
A/India/8942/2012
A/Delhi/1938/2012
A/Delhi/1939/2012
A/Zambia/CZC1/2009
A/South Africa/2/2009
A/Brisbane/10/2010
A/Christchurch/16/2010
A/Tanzania/233/2010
A/Seychelles/106/2009
A/Peru/8109/2009
A/Chile/1174/2009
A/India/2993/2009
A/Yap/2020/2009
A/Bolivia/1263/2009
A/India/3508/2010
A/India/4725/2009
A/India/8489/2009
A/Argentina/19527/2009
A/Nauru/2008/2009
A/Madagascar/10201/2009
A/Castro/12274/2010
A/Puerto Montt/11868/2010
A/Ontario/R255/2011
A/Bolivia/193/2011
A/Uganda/2707/2011
A/South Africa/6/2009
A/Nigeria/4280/2011
A/Fiji/2048/2009
A/Kiribati/2003/2009
A/Simbu/1/2010
A/Samoa/48/2009
A/Paraguay/813/2010
A/Vanimo/7/2010
A/Srinagar/234/2011
A/Srinagar/385/2011
A/Srinagar/04/2011
A/Wisconsin/08/2010
A/India/007/2010
A/India/3725/2010
A/St Petersburg/25/2011
A/Florida/16/2010
A/Pennsylvania/02/2011
A/Alaska/14/2011
A/Maryland/04/2011
A/Mexico/2208/2011
A/South Carolina/02/2010
A/Palau/1/2009
A/California/07/2009
0.001

C

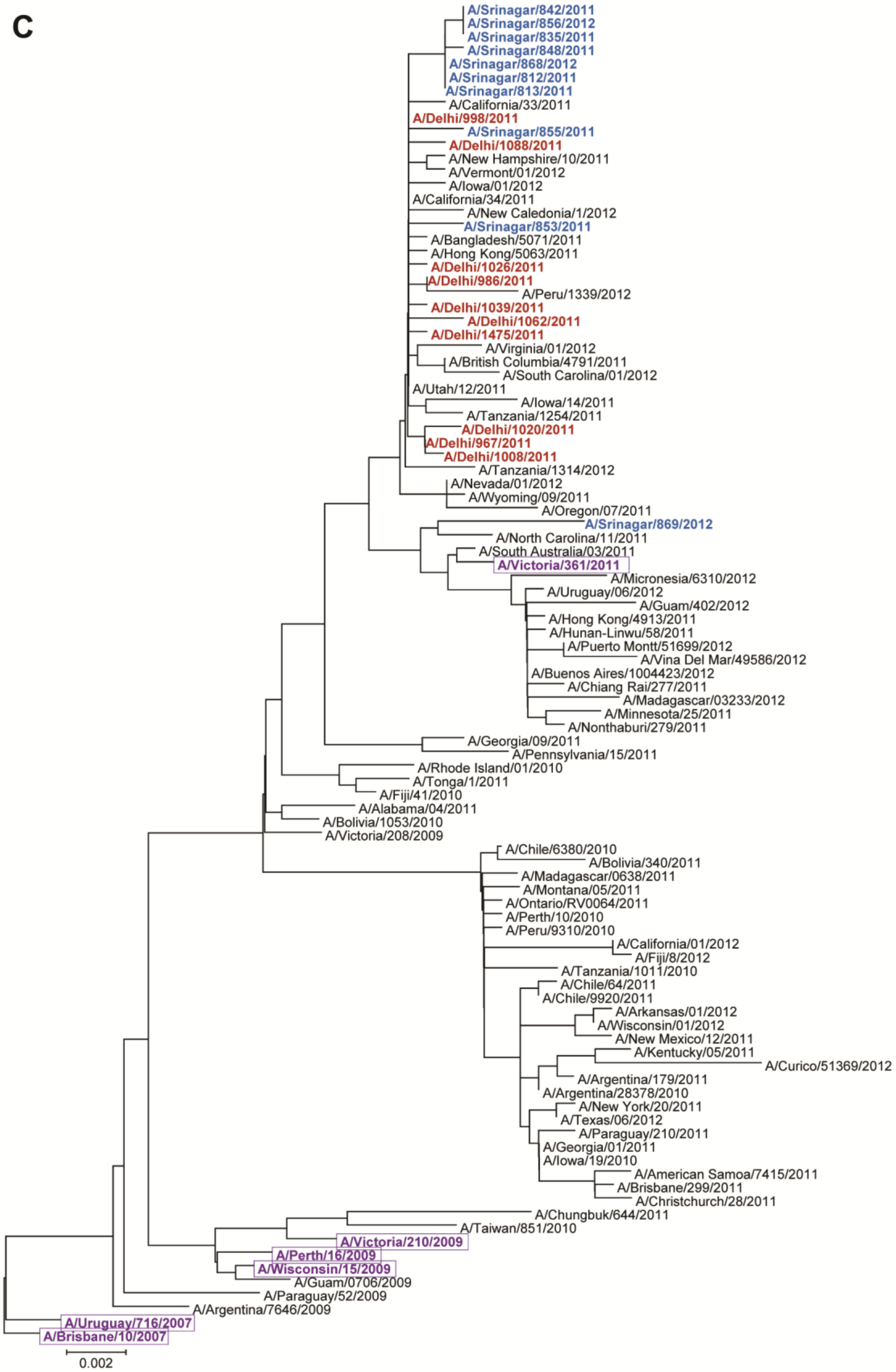
A/Srinagar/842/2011
A/Srinagar/856/2012
A/Srinagar/835/2011
A/Srinagar/848/2011
A/Srinagar/868/2012
A/Srinagar/812/2011
A/Srinagar/813/2011
A/California/33/2011
A/Delhi/998/2011
A/Srinagar/855/2011
A/Delhi/1088/2011
A/New Hampshire/10/2011
A/Vermont/01/2012
A/Iowa/01/2012
A/California/34/2011
A/New Caledonia/1/2012
A/Srinagar/853/2011
A/Bangladesh/5071/2011
A/Hong Kong/5063/2011
A/Delhi/1026/2011
A/Delhi/986/2011
A/Peru/1339/2012
A/Delhi/1039/2011
A/Delhi/1062/2011
A/Delhi/1475/2011
A/Virginia/01/2012
A/British Columbia/4791/2011
A/South Carolina/01/2012
A/Utah/12/2011
A/Iowa/14/2011
A/Tanzania/1254/2011
A/Delhi/1020/2011
A/Delhi/967/2011
A/Delhi/1008/2011
A/Tanzania/1314/2012
A/Nevada/01/2012
A/Wyoming/09/2011
A/Oregon/07/2011
A/Srinagar/869/2012
A/North Carolina/11/2011
A/South Australia/03/2011
A/Victoria/361/2011
A/Micronesia/6310/2012
A/Uruguay/06/2012
A/Guam/402/2012
A/Hong Kong/4913/2011
A/Hunan-Linwu/58/2011
A/Puerto Montt/51699/2012
A/Vina Del Mar/49586/2012
A/Buenos Aires/1004423/2012
A/Chiang Rai/277/2011
A/Madagascar/03233/2012
A/Minnesota/25/2011
A/Nonthaburi/279/2011
A/Georgia/09/2011
A/Pennsylvania/15/2011
A/Rhode Island/01/2010
A/Tonga/1/2011
A/Fiji/41/2010
A/Alabama/04/2011
A/Bolivia/1053/2010
A/Victoria/208/2009
A/Chile/6380/2010
A/Bolivia/340/2011
A/Madagascar/0638/2011
A/Montana/05/2011
A/Ontario/RV0064/2011
A/Perth/10/2010
A/Peru/9310/2010
A/California/01/2012
A/Fiji/8/2012
A/Tanzania/1011/2010
A/Chile/64/2011
A/Chile/9920/2011
A/Arkansas/01/2012
A/Wisconsin/01/2012
A/New Mexico/12/2011
A/Kentucky/05/2011
A/Curico/51369/2012
A/Argentina/179/2011
A/Argentina/28378/2010
A/New York/20/2011
A/Texas/06/2012
A/Paraguay/210/2011
A/Georgia/01/2011
A/Iowa/19/2010
A/American Samoa/7415/2011
A/Brisbane/299/2011
A/Christchurch/28/2011
A/Chungbuk/644/2011
A/Taiwan/851/2010
A/Victoria/210/2009
A/Perth/16/2009
A/Wisconsin/15/2009
A/Guam/0706/2009
A/Paraguay/52/2009
A/Argentina/7646/2009
A/Uruguay/716/2007
A/Brisbane/10/2007
0.002

D

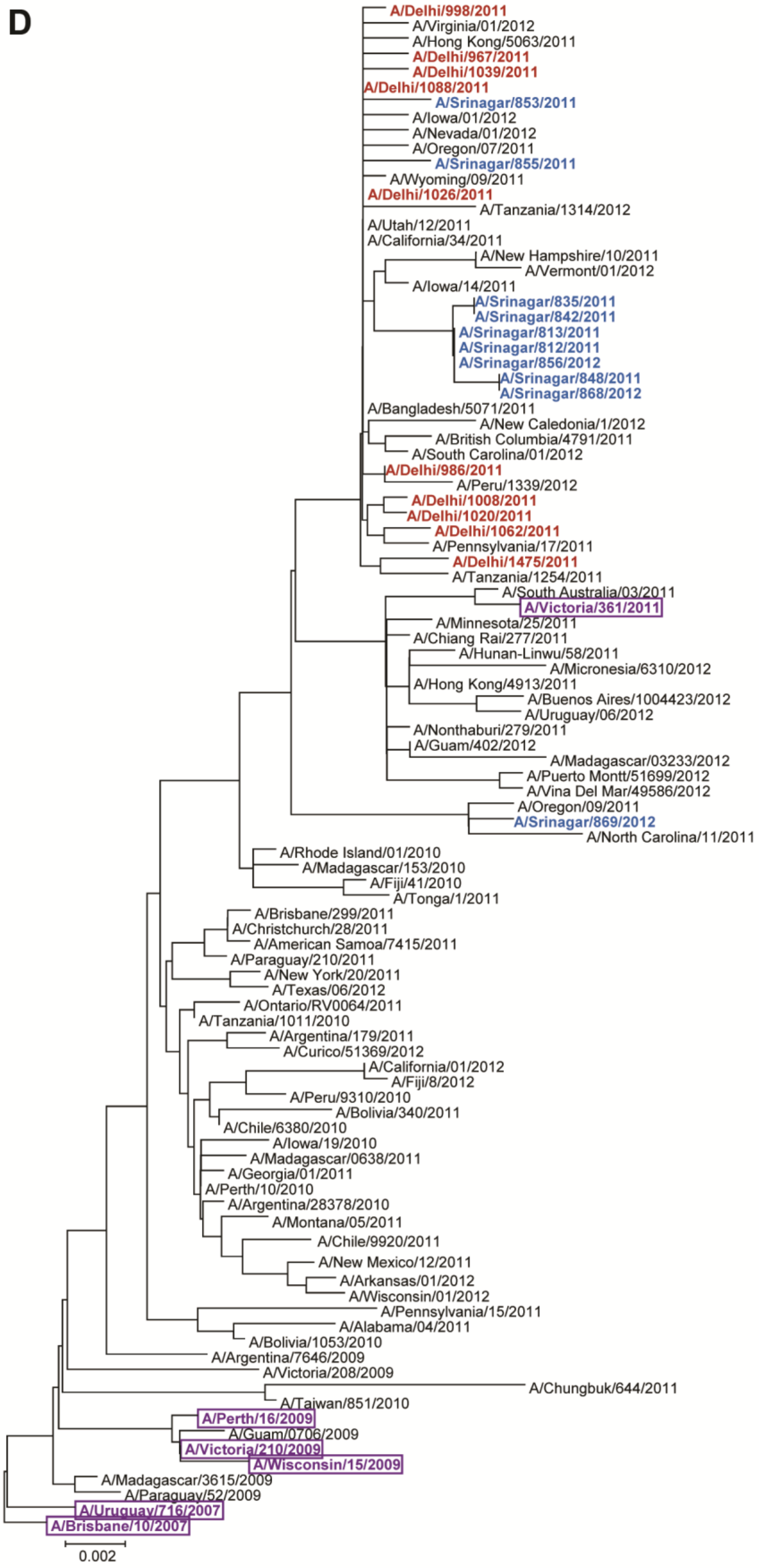
A/Delhi/998/2011
A/Virginia/01/2012
A/Hong Kong/5063/2011
A/Delhi/967/2011
A/Delhi/1039/2011
A/Delhi/1088/2011
A/Srinagar/853/2011
A/Iowa/01/2012
A/Nevada/01/2012
A/Oregon/07/2011
A/Srinagar/855/2011
A/Wyoming/09/2011
A/Delhi/1026/2011
A/Tanzania/1314/2012
A/Utah/12/2011
A/California/34/2011
A/New Hampshire/10/2011
A/Vermont/01/2012
A/Iowa/14/2011
A/Srinagar/835/2011
A/Srinagar/842/2011
A/Srinagar/813/2011
A/Srinagar/812/2011
A/Srinagar/856/2012
A/Srinagar/848/2011
A/Srinagar/868/2012
A/Bangladesh/5071/2011
A/New Caledonia/1/2012
A/British Columbia/4791/2011
A/South Carolina/01/2012
A/Delhi/986/2011
A/Peru/1339/2012
A/Delhi/1008/2011
A/Delhi/1020/2011
A/Delhi/1062/2011
A/Pennsylvania/17/2011
A/Delhi/1475/2011
A/Tanzania/1254/2011
A/South Australia/03/2011
A/Victoria/361/2011
A/Minnesota/25/2011
A/Chiang Rai/277/2011
A/Hunan-Linwu/58/2011
A/Micronesia/6310/2012
A/Hong Kong/4913/2011
A/Buenos Aires/1004423/2012
A/Uruguay/06/2012
A/Nonthaburi/279/2011
A/Guam/402/2012
A/Madagascar/03233/2012
A/Puerto Montt/51699/2012
A/Vina Del Mar/49586/2012
A/Oregon/09/2011
A/Srinagar/869/2012
A/North Carolina/11/2011
A/Rhode Island/01/2010
A/Madagascar/153/2010
A/Fiji/41/2010
A/Tonga/1/2011
A/Brisbane/299/2011
A/Christchurch/28/2011
A/American Samoa/7415/2011
A/Paraguay/210/2011
A/New York/20/2011
A/Texas/06/2012
A/Ontario/RV0064/2011
A/Tanzania/1011/2010
A/Argentina/179/2011
A/Curico/51369/2012
A/California/01/2012
A/Fiji/8/2012
A/Peru/9310/2010
A/Bolivia/340/2011
A/Chile/6380/2010
A/Iowa/19/2010
A/Madagascar/0638/2011
A/Georgia/01/2011
A/Perth/10/2010
A/Argentina/28378/2010
A/Montana/05/2011
A/Chile/9920/2011
A/New Mexico/12/2011
A/Arkansas/01/2012
A/Wisconsin/01/2012
A/Pennsylvania/15/2011
A/Alabama/04/2011
A/Bolivia/1053/2010
A/Argentina/7646/2009
A/Victoria/208/2009
A/Chungbuk/644/2011
A/Taiwan/851/2010
A/Perth/16/2009
A/Guam/0706/2009
A/Victoria/210/2009
A/Wisconsin/15/2009
A/Madagascar/3615/2009
A/Paraguay/52/2009
A/Uruguay/716/2007
A/Brisbane/10/2007
0.002

E

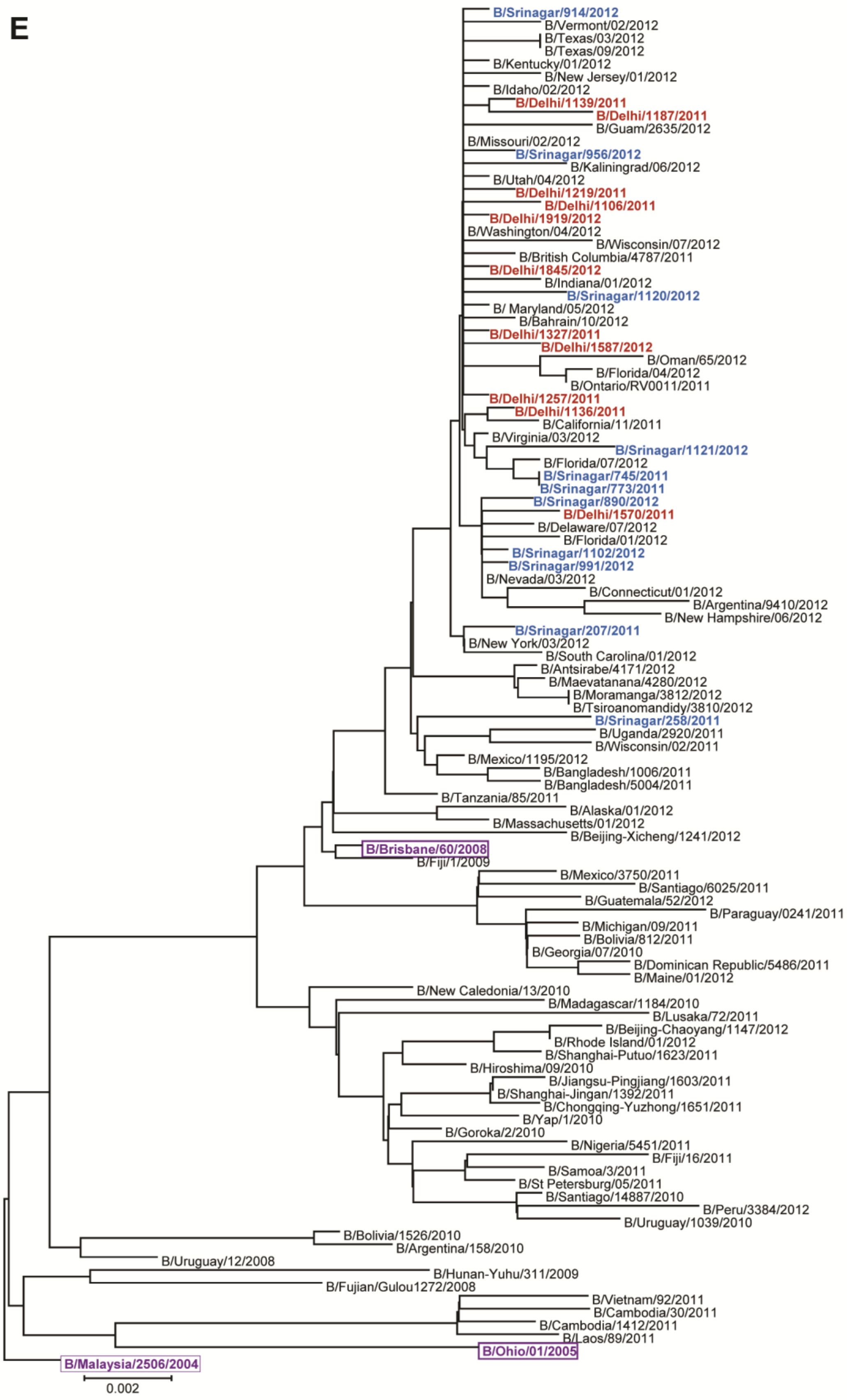
B/Srinagar/914/2012
B/Vermont/02/2012
B/Texas/03/2012
B/Texas/09/2012
B/Kentucky/01/2012
B/New Jersey/01/2012
B/Idaho/02/2012
B/Delhi/1139/2011
B/Delhi/1187/2011
B/Guam/2635/2012
B/Missouri/02/2012
B/Srinagar/956/2012
B/Kaliningrad/06/2012
B/Utah/04/2012
B/Delhi/1219/2011
B/Delhi/1106/2011
B/Delhi/1919/2012
B/Washington/04/2012
B/Wisconsin/07/2012
B/British Columbia/4787/2011
B/Delhi/1845/2012
B/Indiana/01/2012
B/Srinagar/1120/2012
B/ Maryland/05/2012
B/Bahrain/10/2012
B/Delhi/1327/2011
B/Delhi/1587/2012
B/Oman/65/2012
B/Florida/04/2012
B/Ontario/RV0011/2011
B/Delhi/1257/2011
B/Delhi/1136/2011
B/California/11/2011
B/Virginia/03/2012
B/Srinagar/1121/2012
B/Florida/07/2012
B/Srinagar/745/2011
B/Srinagar/773/2011
B/Srinagar/890/2012
B/Delhi/1570/2011
B/Delaware/07/2012
B/Florida/01/2012
B/Srinagar/1102/2012
B/Srinagar/991/2012
B/Nevada/03/2012
B/Connecticut/01/2012
B/Argentina/9410/2012
B/New Hampshire/06/2012
B/Srinagar/207/2011
B/New York/03/2012
B/South Carolina/01/2012
B/Antsirabe/4171/2012
B/Maevatanana/4280/2012
B/Moramanga/3812/2012
B/Tsiroanomandidy/3810/2012
B/Srinagar/258/2011
B/Uganda/2920/2011
B/Wisconsin/02/2011
B/Mexico/1195/2012
B/Bangladesh/1006/2011
B/Bangladesh/5004/2011
B/Tanzania/85/2011
B/Alaska/01/2012
B/Massachusetts/01/2012
B/Beijing-Xicheng/1241/2012
B/Brisbane/60/2008
B/Fiji/1/2009
B/Mexico/3750/2011
B/Santiago/6025/2011
B/Guatemala/52/2012
B/Paraguay/0241/2011
B/Michigan/09/2011
B/Bolivia/812/2011
B/Georgia/07/2010
B/Dominican Republic/5486/2011
B/Maine/01/2012
B/New Caledonia/13/2010
B/Madagascar/1184/2010
B/Lusaka/72/2011
B/Beijing-Chaoyang/1147/2012
B/Rhode Island/01/2012
B/Shanghai-Putuo/1623/2011
B/Hiroshima/09/2010
B/Jiangsu-Pingjiang/1603/2011
B/Shanghai-Jingan/1392/2011
B/Chongqing-Yuzhong/1651/2011
B/Yap/1/2010
B/Goroka/2/2010
B/Nigeria/5451/2011
B/Fiji/16/2011
B/Samoa/3/2011
B/St Petersburg/05/2011
B/Santiago/14887/2010
B/Peru/3384/2012
B/Uruguay/1039/2010
B/Bolivia/1526/2010
B/Argentina/158/2010
B/Uruguay/12/2008
B/Hunan-Yuhu/311/2009
B/Fujian/Gulou1272/2008
B/Vietnam/92/2011
B/Cambodia/30/2011
B/Cambodia/1412/2011
B/Laos/89/2011
B/Ohio/01/2005
B/Malaysia/2506/2004
0.002

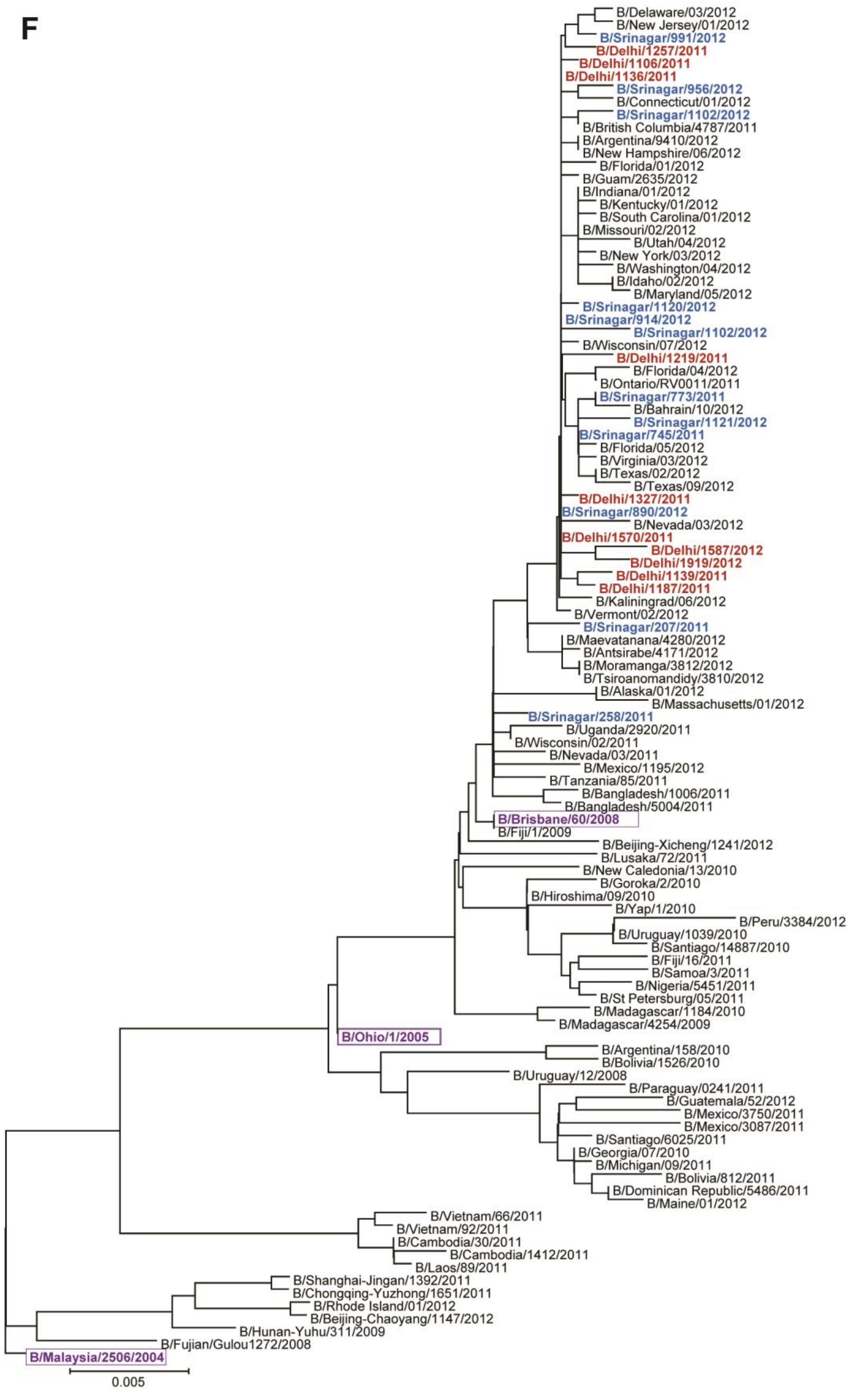
F
B/Delaware/03/2012
B/New Jersey/01/2012
B/Srinagar/991/2012
B/Delhi/1257/2011
B/Delhi/1106/2011
B/Delhi/1136/2011
B/Srinagar/956/2012
B/Connecticut/01/2012
B/Srinagar/1102/2012
B/British Columbia/4787/2011
B/Argentina/9410/2012
B/New Hampshire/06/2012
B/Florida/01/2012
B/Guam/2635/2012
B/Indiana/01/2012
B/Kentucky/01/2012
B/South Carolina/01/2012
B/Missouri/02/2012
B/Utah/04/2012
B/New York/03/2012
B/Washington/04/2012
B/Idaho/02/2012
B/Maryland/05/2012
B/Srinagar/1120/2012
B/Srinagar/914/2012
B/Srinagar/1102/2012
B/Wisconsin/07/2012
B/Delhi/1219/2011
B/Florida/04/2012
B/Ontario/RV0011/2011
B/Srinagar/773/2011
B/Bahrain/10/2012
B/Srinagar/1121/2012
B/Srinagar/745/2011
B/Florida/05/2012
B/Virginia/03/2012
B/Texas/02/2012
B/Texas/09/2012
B/Delhi/1327/2011
B/Srinagar/890/2012
B/Nevada/03/2012
B/Delhi/1570/2011
B/Delhi/1587/2012
B/Delhi/1919/2012
B/Delhi/1139/2011
B/Delhi/1187/2011
B/Kaliningrad/06/2012
B/Vermont/02/2012
B/Srinagar/207/2011
B/Maevatanana/4280/2012
B/Antsirabe/4171/2012
B/Moramanga/3812/2012
B/Tsiroanomandidy/3810/2012
B/Alaska/01/2012
B/Massachusetts/01/2012
B/Srinagar/258/2011
B/Uganda/2920/2011
B/Wisconsin/02/2011
B/Nevada/03/2011
B/Mexico/1195/2012
B/Tanzania/85/2011
B/Bangladesh/1006/2011
B/Bangladesh/5004/2011
B/Brisbane/60/2008
B/Fiji/1/2009
B/Beijing-Xicheng/1241/2012
B/Lusaka/72/2011
B/New Caledonia/13/2010
B/Goroka/2/2010
B/Hiroshima/09/2010
B/Yap/1/2010
B/Peru/3384/2012
B/Uruguay/1039/2010
B/Santiago/14887/2010
B/Fiji/16/2011
B/Samoa/3/2011
B/Nigeria/5451/2011
B/St Petersburg/05/2011
B/Madagascar/1184/2010
B/Madagascar/4254/2009
B/Ohio/1/2005
B/Argentina/158/2010
B/Bolivia/1526/2010
B/Uruguay/12/2008
B/Paraguay/0241/2011
B/Guatemala/52/2012
B/Mexico/3750/2011
B/Mexico/3087/2011
B/Santiago/6025/2011
B/Georgia/07/2010
B/Michigan/09/2011
B/Bolivia/812/2011
B/Dominican Republic/5486/2011
B/Maine/01/2012
B/Vietnam/66/2011
B/Vietnam/92/2011
B/Cambodia/30/2011
B/Cambodia/1412/2011
B/Laos/89/2011
B/Shanghai-Jingan/1392/2011
B/Chongqing-Yuzhong/1651/2011
B/Rhode Island/01/2012
B/Beijing-Chaoyang/1147/2012
B/Hunan-Yuhu/311/2009
B/Fujian/Gulou1272/2008
B/Malaysia/2506/2004
0.005

G

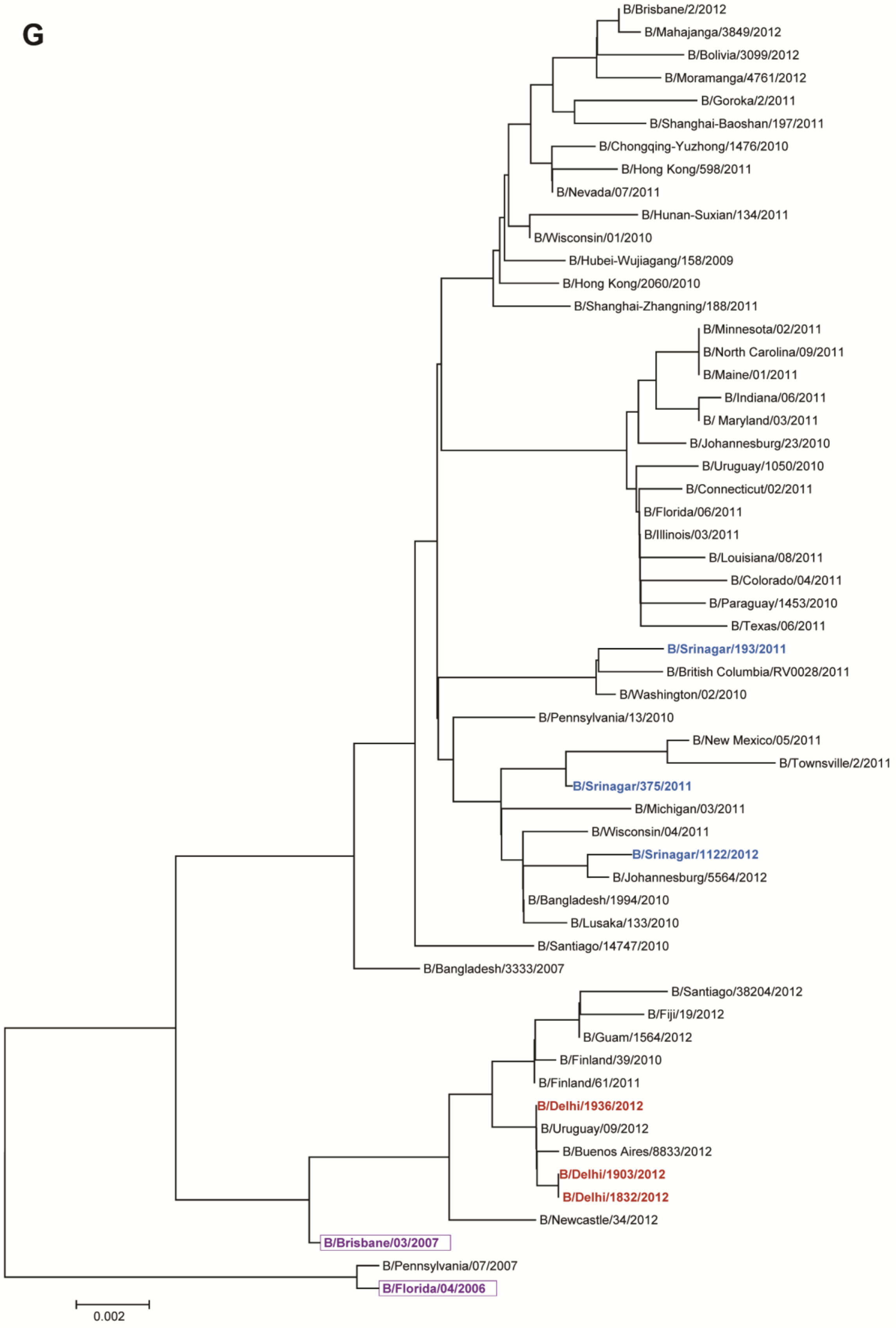

B/Brisbane/2/2012
B/Mahajanga/3849/2012
B/Bolivia/3099/2012
B/Moramanga/4761/2012
B/Goroka/2/2011
B/Shanghai-Baoshan/197/2011
B/Chongqing-Yuzhong/1476/2010
B/Hong Kong/598/2011
B/Nevada/07/2011
B/Hunan-Suxian/134/2011
B/Wisconsin/01/2010
B/Hubei-Wujiagang/158/2009
B/Hong Kong/2060/2010
B/Shanghai-Zhangning/188/2011
B/Minnesota/02/2011
B/North Carolina/09/2011
B/Maine/01/2011
B/Indiana/06/2011
B/ Maryland/03/2011
B/Johannesburg/23/2010
B/Uruguay/1050/2010
B/Connecticut/02/2011
B/Florida/06/2011
B/Illinois/03/2011
B/Louisiana/08/2011
B/Colorado/04/2011
B/Paraguay/1453/2010
B/Texas/06/2011
B/Srinagar/193/2011
B/British Columbia/RV0028/2011
B/Washington/02/2010
B/Pennsylvania/13/2010
B/New Mexico/05/2011
B/Townsville/2/2011
B/Srinagar/375/2011
B/Michigan/03/2011
B/Wisconsin/04/2011
B/Srinagar/1122/2012
B/Johannesburg/5564/2012
B/Bangladesh/1994/2010
B/Lusaka/133/2010
B/Santiago/14747/2010
B/Bangladesh/3333/2007
B/Santiago/38204/2012
B/Fiji/19/2012
B/Guam/1564/2012
B/Finland/39/2010
B/Finland/61/2011
B/Delhi/1936/2012
B/Uruguay/09/2012
B/Buenos Aires/8833/2012
B/Delhi/1903/2012
B/Delhi/1832/2012
B/Newcastle/34/2012
B/Brisbane/03/2007
B/Pennsylvania/07/2007
B/Florida/04/2006
0.002

H

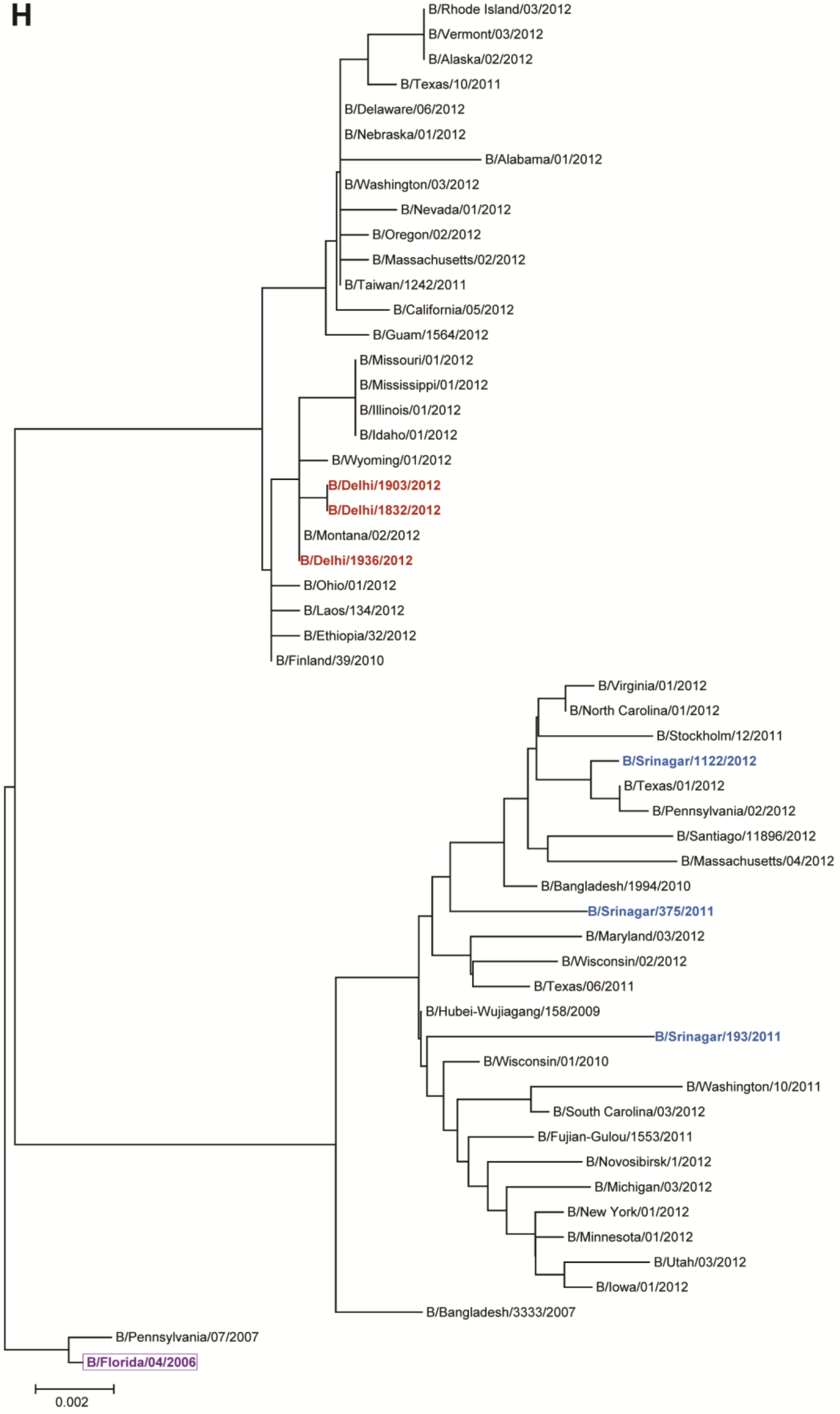
B/Rhode Island/03/2012
B/Vermont/03/2012
B/Alaska/02/2012
B/Texas/10/2011
B/Delaware/06/2012
B/Nebraska/01/2012
B/Alabama/01/2012
B/Washington/03/2012
B/Nevada/01/2012
B/Oregon/02/2012
B/Massachusetts/02/2012
B/Taiwan/1242/2011
B/California/05/2012
B/Guam/1564/2012
B/Missouri/01/2012
B/Mississippi/01/2012
B/Illinois/01/2012
B/Idaho/01/2012
B/Wyoming/01/2012
B/Delhi/1903/2012
B/Delhi/1832/2012
B/Montana/02/2012
B/Delhi/1936/2012
B/Ohio/01/2012
B/Laos/134/2012
B/Ethiopia/32/2012
B/Finland/39/2010
B/Virginia/01/2012
B/North Carolina/01/2012
B/Stockholm/12/2011
B/Srinagar/1122/2012
B/Texas/01/2012
B/Pennsylvania/02/2012
B/Santiago/11896/2012
B/Massachusetts/04/2012
B/Bangladesh/1994/2010
B/Srinagar/375/2011
B/Maryland/03/2012
B/Wisconsin/02/2012
B/Texas/06/2011
B/Hubei-Wujiagang/158/2009
B/Srinagar/193/2011
B/Wisconsin/01/2010
B/Washington/10/2011
B/South Carolina/03/2012
B/Fujian-Gulou/1553/2011
B/Novosibirsk/1/2012
B/Michigan/03/2012
B/New York/01/2012
B/Minnesota/01/2012
B/Utah/03/2012
B/Iowa/01/2012
B/Bangladesh/3333/2007
B/Pennsylvania/07/2007
B/Florida/04/2006
0.002

Technical Appendix Figure 2. World Health Organization recommended vaccines by season and hemisphere, 2006–2014.

Northern Hemisphere				Southern Hemisphere		
Season	Recommended Strain	alternates	Subtype	Season	Recommended Strain	alternates
2006-07	Wisconsin/67/2005	Hiroshima/52/2005	**H3N2**	2007	Wisconsin/67/2005	Hiroshima/52/2005
	New Caledonia/20/99		**H1N1**		New Caledonia/20/99	
	Malaysia/2506/2004	Ohio/1/2005	**B Victoria**		Malaysia/2506/2004	
			**B Yamagata**			
2007-08	Wisconsin/67/2005	Hiroshima/52/2005	**H3N2**	2008	Brisbane/10/2007	
	Solomon Islands/1513/2006		**H1N1**		Solomon Islands/1513/2006	
	Malaysia/2506/2004		**B Victoria**			
			**B Yamagata**		Florida/4/2006	
2008-09	Brisbane/10/2007		**H3N2**	2009	Brisbane/10/2007	Uruguay/716/2007
	Brisbane/59/2007		**H1N1**		Brisbane/59/2007	South Dakota/6/2007
			**B Victoria**			
	Florida/4/2006		**B Yamagata**		Florida/4/2006	Brisbane/3/2007
2009-10	Brisbane/10/2007	Uruguay/716/2007	**H3N2**	2010	Perth/16/2009	
	Brisbane/59/2007	South Dakota/6/2007	**H1N1**	H1pdm	California/7/2009	
	Brisbane/60/2008	Brisbane/33/2008	**B Victoria**		Brisbane/60/2008	
			**B Yamagata**			
2010-11	Perth/16/2009	Wisconsin/15/2009	**H3N2**	2011	Perth/16/2009	Wisconsin/15/2009<br>Victoria/210/2009
	California/7/2009		**H1N1pdm**		California/7/2009	
	Brisbane/60/2008		**B Victoria**		Brisbane/60/2008	
			**B Yamagata**			
2011-12	Perth/16/2009		**H3N2**	2012	Perth/16/2009	
	California/7/2009		**H1N1pdm**		California/7/2009	
	Brisbane/60/2008		**B Victoria**		Brisbane/60/2008	
			**B Yamagata**			
2012-13	Victoria/361/2011		**H3N2**	2013	Victoria/361/2011	Brisbane/6/2012<br>Ohio/2/2012<br>Maryland/2/2012<br>South Australia/30/2012<br>Brisbane/1/2012<br>Christ Church/16/2010
	California/7/2009		**H1N1pdm**		California/7/2009	
			**B Victoria**		Brisbane/60/2008	
	Wisconsin/1/2010		**B Yamagata**			
2013-14	Victoria/361/2011	Texas/50/2012	**H3N2**	2014	Texas/50/2012	
	California/7/2009	Christ Church/10/2010	**H1N1pdm**		California/7/2009	Christ Church/10/2010
	Brisbane/60/2008		**B Victoria**		Brisbane/60/2008	
	Massachussetts/2/2012		**B Yamagata**		Massachussetts/2/2012	
